# Increased cortisol levels caused by acute resistance physical exercise impair memory and learning ability

**DOI:** 10.7717/peerj.13000

**Published:** 2022-03-23

**Authors:** José-Luis Bermejo, Raúl Valldecabres, Israel Villarrasa-Sapiña, Gonzalo Monfort-Torres, Adrià Marco-Ahulló, Bruno Ribeiro Do Couto

**Affiliations:** 1 Department of Physical Activity and Sport Sciences, University of Valencia, Valencia, Spain; 2 Human Movement Analysis Research Group - HuMAG (GIUV2016-306), University of Valencia, Valencia, Spain; 3 Faculty of Education, Valencia International University - VIU, Valencia, Spain; 4 Universidad Internacional de la Rioja - UNIR, Logroño, Spain; 5 Unidad de Educación, Florida Universitaria, Catarroja, Valencia, Spain; 6 Departamento de Neuropsicología, metodología, psicología social y básica. Facultad de Psicología. Universidad Católica de Valencia; 7 Institute of Biomedical Research of Murcia, Virgen de la Arrixaca University Hospital, University of Murcia, Murcia, Spain; 8 Department of Human Anatomy and Psychobiology, Faculty of Psychology, University of Murcia, Murcia, Spain

**Keywords:** Acute exercise, Cortisol, Cognitive performance, Memory, Learning

## Abstract

Acute physical exercise works as an activator of the responses of the human organism to stress. This is based on the activation of the hypothalamic–pituitary–adrenal (HPA) axis, affecting physical, physiological and psychological levels. This study aimed to analyse the effects of a single bout of high-intensity resistance exercise on cognitive-behavioural responses: visuo-spatial path learning and memory, as well as physiological responses (salivary cortisol levels). Nineteen healthy male military-trained powerlifting subjects were tested in a within-subject design on two experimental days with an interval of 48 h. The stress and cognitive variables were measured by cortisol levels and Ruff–Light trail-learning test (RULIT) test scores, respectively. The results showed the immediate influence of acute exercise on cortisol, with significantly higher cortisol levels found in subjects after completion of the acute resistance exercise. In addition, this study found a significant deterioration of memory and learning ability after a dose of intense resistance exercise. In conclusion, the study highlights the relative effects of resistance exercise on cortisol and cognitive performance depending on the intensity and type of the exercise, the moment of measurement and the cerebral areas implicated.

## Introduction

Recently, most research on the effects of physical exercise has focused on the adult population and the effect of physical activity on slowing down the ageing process, physical abilities (*e.g*. muscular strength) and/or cognitive functions (*e.g*. learning and memory) since a lack of physical exercise is associated with disability and decreased life-span, considerably affecting quality of life in the elderly (Murray et al., 2013; Herold et al., 2019).

The ability to orient oneself with maps or by recognising features in the environment is an important mental activity in daily life that underlies both cognition and behaviour (Taverniers et al., 2011). Moreover, it gains practical salience in occupations like the military, police and emergency services, for whose employees path learning and wayfinding under intense stress are commonly required skills with a profound operational effect (Branaghan et al., 2010).

Attempts have been made to establish the effects of physical exercise, whether chronic or acute, on various factors, such as sleep, mental health, physiological variables (*e.g*. glucose absorption) or cognitive variables (*e.g*. memory or learning; Esteves et al., 2009; Anderson & Shivakumar, 2013; Hötting & Röder, 2013; Röhling et al., 2016). In relation to physical exercise and its effects on cognition, it is possible to distinguish between those effects induced by specific physical exercise after a single session and those based on chronic adaptation. Acute exercise has reversible short-term effects on the cognitive system (Chang et al., 2012; McMorris et al., 2009). By contrast, those activities planned through structured training programmes tend to have more lasting effects on different brain structures and functions (Chaddock et al., 2011; Erickson et al., 2011).

Results from studies on the effects of high-intensity (acute) exercise on cognition vary; different studies have shown that performance improves (Bermejo et al., 2018), does not change (Mierau et al., 2014), declines (Moore et al., 2012) or may even reverse into an impairment when exercise is prolonged and/or the individual reaches exhaustion (Sudo et al., 2017).

Recent work (Roig et al., 2016; Loprinzi et al., 2018; Haynes et al., 2019) demonstrates that acute aerobic exercise can enhance short- and long-term episodic memory function, as well as semantic memory (Smith et al., 2013). In this sense, most of these studies have examined the effects of aerobic exercise, although it has been shown that acute resistance exercise may also enhance cognition, particularly inhibitory control (Soga et al., 2018), but more research is needed on this exercise modality (Loprinzi, Loenneke & Storm, 2021).

Although both aerobic and resistance exercise have been shown to favourably influence cerebral blood flow and neurogenesis (Loprinzi et al., 2020), resistance exercise differs from aerobic exercise in its physiological demands (*e.g*. cardiovascular, musculoskeletal, metabolic; Fletcher et al., 2013) and influencing mechanisms in cognitive processes (*e.g*. structural brain changes, increased neural excitability; Loprinzi et al., 2020), which may have a contrary effect on its cognitive effects.

Some of these differential mechanisms are suggested in a review by Loprinzi et al. (2020), while aerobic exercise increases hippocampal levels of brain-derived neurotrophic factor (BDNF) and Tropomyosin receptor kinase B (TrkB), protein kinases and glutamatergic proteins. In addition, resistance exercise supports a reduction in IL-6 (Interleukin-6), which inhibits N-Methyl-D-aspartate (NMDA) activity. Moreover, the increased IGF-1 (insulin-like growth factor 1) production is one of the most important hormones for growth and development in humans (Sonntag, Ramsey & Carter, 2005) and a recognised candidate factor that specifically connects resistance-exercise training and cognition (Chang et al., 2011).

Regarding the relationship of resistance exercise and cognitive functions, we must mention one of the most notable known hypotheses: “common cause hypothesis,” which proposes that cognition and muscle strength may share brain regions and networks (Christensen et al., 2001). This would mean that a bout of acute resistance exercise could affect cognitive functions (Kim et al., 2018; Liu et al., 2019). The limited literature that has examined the effects of a bout of acute resistance training has found contradictory results. Most of these studies claim that acute resistance exercise has no effect on memory (Weinberg et al., 2014; Loprinzi, Loenneke & Storm, 2021) or that it may impair memory function (Loprinzi et al., 2020).

All things considered, the aforementioned studies point to exercise intensity (high or moderate) as an important factor in the exercise–cognition relationship (Tomporowski, 2003). This diversity of results does not necessarily challenge the assumption that there is a positive relationship between acute exercise and cognition but rather demonstrates that this relationship is complex and sensitive to multiple factors (Lambourne & Tomporowski, 2010). These factors include exercise intensity (Tomporowski, 2003), physical condition (Ludyga et al., 2016), gender (Barha et al., 2017), exercise type, time of assessment and the cognitive task tested (Lambourne & Tomporowski, 2010), among others. In addition, the time between the cessation of exercise and the evaluation of cognitive functions is another crucial variable due to the transience of the psychophysiological effects of acute exercise (Crabbe & Dishman, 2004).

Several mechanisms resulting from a further increase in the duration (beyond 1 h) of prolonged exercise, however, such as dehydration or hypoglycaemia, have been linked to central nervous system fatigue and decreased cognitive performance (Brisswalter, Collardeau & René, 2002). This is partly due to the fact that acute physical exercise works as an activator of the human organism’s responses to stress, based on the activation of the hypothalamic–pituitary–adrenal (HPA) axis, affecting the physical, physiological and psychological levels (Heijnen et al., 2016) and causing a substantial increase in the circulating concentrations of cortisol (Kraemer et al., 1999). This increase in circulating cortisol may be critical to cognitive performance (Almela et al., 2012; Quesada et al., 2012). In addition, we must bear in mind that most cortisol receptors are found in the hippocampus, an essential area for learning and memory (Elzinga & Bremner, 2002; Shin & Liberzon, 2010). The relationship between acute stress and cognition, however, may not always be linear; both high and low levels of circulating glucocorticoids can impair memory performance compared to more moderate levels (Lupien et al., 2002).

Research has shown that human response to stress, based on the secretion of glucocorticoids, can modulate learning and memory (by facilitating or impairing them; Het, Ramlow & Wolf, 2005; Smeets et al., 2008; Wolf, 2009). Most research on acute physical exercise and its effects has studied protocols based on short or long periods of aerobic exercise (Bermejo et al., 2019; Chang & Etnier, 2009; Pontifex et al., 2009). Research examining the effects of acute resistance exercise on cognitive performance, however, is limited (Chang et al., 2014), and substantially more research is needed to facilitate the understanding of whether acute resistance exercise benefits or impairs such performance. Above all, we know that there are acute hormonal responses to a single bout of heavy resistance exercise when the intensities of the load cause exhaustion. Moreover, there is an even greater increase in stress hormones (*e.g*. cortisol), which can reach a tenfold increase from the base level, which in turn quickly leads to a state of psychophysical overload (Lehmann et al., 1980).

In general, these findings may have important implications for exercise prescription purposes. For example, these implications include the timing of exercise and the duration of the recovery period to try to optimise cognitive functions (Loprinzi et al., 2020) and in particular may provide individuals with evidence of the efficacy of other modalities of exercise, not as well studied, such as, in our case, resistance exercise.

Considering all of the above, the authors hypothesise that cognitive performance declines after resistance-induced stress when it coincides with peak cortisol. To confirm or deny this hypothesis, this study aimed to elucidate the effects of a single bout of acute resistance exercise on cognitive behavioural responses: visuo-spatial path learning and memory (working memory and delayed recall) and physiological (salivary cortisol levels).

## Materials and Methods

### Participants

To determine the appropriate sample size for this study, a preliminary power analysis was conducted using the freely available software G*Power 3.1.9 (University of Düsseldorf, Düsseldorf, Germany). The effect-size calculation was based on recent reviews of acute stressors and cortisol responses (Dickerson & Kemeny, 2004) and the effect of acute exercise on cognitive performances (Chang et al., 2012). The optimal sample size of nineteen participants was calculated by fixing the probability of a type 1 error at an alpha of 0.05 to yield a 0.80 power for an effect size of 0.28. Nineteen healthy male military-trained powerlifting subjects (mean (SE); age: 32.5 (0.96) years; weight: 78.02 (1.63) kg; height: 175.35 (2.5) cm) were recruited to participate in this study. All subjects were physically active (at least 5 days/week of physical activity practice), could lift at least 1.5 times their body weight during the half-squat exercise and had no history of any neurological or psychiatric diseases, drug abuse or medication intake that might influence their results (Loprinzi, Loenneke & Storm, 2021). In addition, the participants were experienced map readers (12.75 (6.6) years of practice) and were chosen because they were able to repeat the same cognitive task (*e.g*. visuo-spatial path learning) in a similar way within two time periods. The sample was randomly selected from the subjects who met the inclusion criteria (www.random.org).

The subjects gave their written informed consent before participating in the study. The protocols used in this research work received ethical clearance from the University of Valencia’s Ethical Committee (Ref. H1402563451425). These protocols also met the requirements set out in the Declaration of Helsinki, 1975, subsequently reviewed in 2008.

### Procedure

Participants were tested in a within-subject design on two experimental days with an interval of 48 h. On day 1, a control condition Ruff–Light trail-learning test (RULIT) was performed, as well as an incremental load protocol to reach the 1RM (one-repetition maximum) and a force-speed curve in a half-squat position (1RMHS) to determinate the Pmax load (the load that maximises power output and which has been shown to elicit the greatest adaptations in muscular power; García-Ramos et al., 2016). Day 2 consisted of a squat induced-stress and cognitive task session. To exclude confounding effects due to circadian cortisol variations (Nicolson, 2008) and circadian peak force rhythm (Gabriel & Zierath, 2019), all testing took place in the afternoon between 14:00 and 18:00 h.

Before data acquisition, researchers informed the participants of the protocol to be performed. Participants then gave their consent to join in the study. In this session, participants were instructed to not take any stimulants less than 24 h before the study (*e.g*. coffee).

#### Day 1: Familiarisation day

After their arrival at the laboratory, participants were fitted with a Polar M400 heart rate monitor (Polar Electro Ltd., Kempele, Finland). Subsequently, the participants completed a relaxation phase on a stretcher, and their breath was monitored with a metronome marking of 40 beats/min for 10 min. Next, participants completed the cognitive task, the Ruff–Light trail-learning test (RULIT), in order to familiarise themselves with the task. Immediately afterwards, an incremental load protocol for calculating 1RMHS and Pmax were performed, as previously described in García-Ramos et al. (2016). The kinematic parameters for each repetition were calculated using a dynamic measurement system (T-Force System; Ergotech, Murcia, Spain).

#### Day 2: Experimental day

In Session 2 (48 h after Session 1), all participants were fitted with a heart rate Polar M400 and repeated the relaxation phase. Each participant performed a fatigue protocol: two sets of half-squats for each of the three loading conditions (Pmax, Pmax −15% and Pmax +15%) (García-Ramos et al., 2016), totalling six sets. Each set was performed to failure or a maximum of 20 repetitions. All participants performed the half-squat using these loads in increasing order from lightest to heaviest, and all repetitions were executed as quickly as possible. The recovery time between sets of the same load was 1 min and between sets of different loads was 3 min (Mayhew, Thyfault & Koch, 2005).

**Figure 1:**
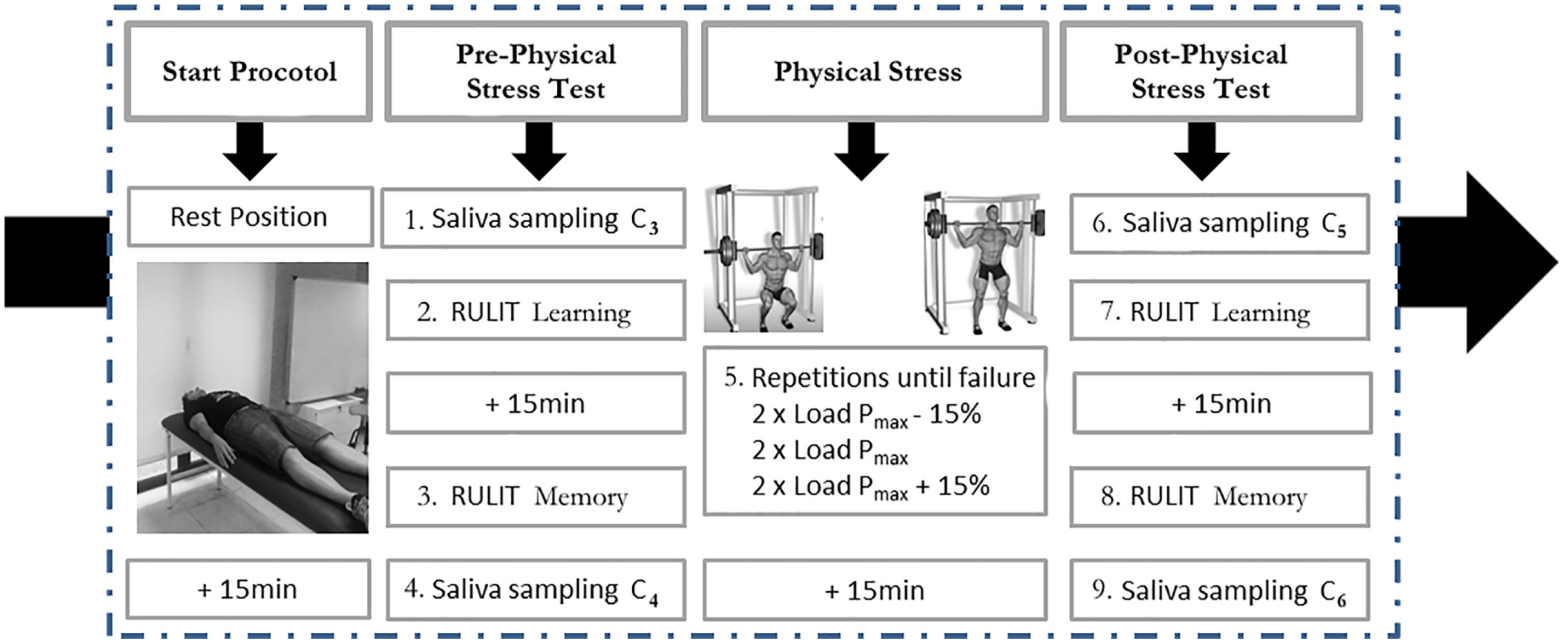
Description of protocol study.

During this session, the cognitive performance of the participants was evaluated by the RULIT to measure learning and memory. Cognitive functions before and after 15 min of physical stress were evaluated, coinciding with the expected highest cortisol concentrations (Nicolson, 2008). After completing both cognitive tasks, cortisol (C) was measured. Figure 1 depicts the experimental setup.

### Measures and materials

#### Saliva sampling and cortisol analysis

During day 2 (the experimental day), four salivary samples were collected according to the criteria established by Chatterton et al. (1997):

- C3 (after participants finished the relaxation phase),
- C4 (15 min after the cognitive tasks and before the physical stress exercise),
- C5 (15 min after this acute muscle exercise) and
- C6 (15 min after completing the cognitive tasks).

On day 1 (the familiarisation day), two salivary samples were collected according to the same criteria:

- C1 (after participants finished the relaxation phase) and
- C2 (15 min after the cognitive tasks).

Saliva samples were taken, and cortisol analysis was performed as previously described in Bermejo et al. (2019).

#### Ruff–Light trail-learning test

Visuo-spatial learning and memory were measured with an adapted military version (Taverniers et al., 2011) of the Ruff–Light trail-learning test (RULIT; Ruff, Light & Parker, 1996). The military-adapted RULIT stimulus card resembled a mock city plan that simulated reconnaissance in built-up areas. In this activity, the participant is asked to learn a specific trail by tracing with an index finger from a START circle to an END circle. From each circle along the way, the person has 2–4 choices for the next circle. At each point (step) along the trail, the researcher informs the person whether they have made a correct or incorrect choice. If their choice was correct, the participant can proceed to the next step; if incorrect, the participant goes back to the previous position on the trail and tries again until the correct choice is made. Successive trials are given until the respondent has gone through the 15-step trail 10 times or until the trail can be recalled without error in two consecutive trials. For more details, see Taverniers et al. (2011) figure.

The RULIT learning and memory test variables evaluated were (*e.g*. Ruff, Light & Parker, 1996):

For learning:
(1) total correct scores, trials 2–10: total number of correct steps (of 15 possible) in the nine trials. Once the task was learned, the 15 steps of each of the unused trials were counted.
(2) total step errors, trials 2–10: total number of erroneous steps in the nine trials.
(3) number of trials to completion: number of trials required to learn the task (9 out of 10 possible). The first trial was not taken into account, as it was the result of chance.
(4) Performance = $\displaystyle{{correct\ scores - errors} \over {number\ of\ trials\ to\ completion}}$

For memory:
(5) Successes followed (working memory): the correct scores followed before and after the first error, to establish which part of the map route was best memorised and see how the memorised steps increased (7 ± 2, Miller’s magic number; Miller, 1956).
(6) Delayed recall: 15 min after taking the learning test, the participants repeated the test to evaluate their retention capacity. Memory was evaluated by the number of total correct score successes until the first mistake in a unique opportunity (just before C4 and C6 sampling).

The participants were each given one of three maps of the same level of difficulty (*i.e*. the same number of changes of direction).

### Statistical analysis

The statistical analysis was performed using SPSS 21 for Windows (IBM Corporation, Armonk, NY, USA). First, we applied descriptive statistics to calculate the mean and median as measures of central tendency and the standard deviation and interquartile range as measures of dispersion. The assumption of normality was then checked by means of Kolmogorov–Smirnoff test. In the case of cortisol, a parametric analysis was applied. Concretely, a *T*-test was applied on the control day and before the acute exercise (C1 *vs*. C3 and C2 *vs*. C4) to determine the immediate influence of acute exercise on cortisol (C5 *vs*. C4 and C6).

Related to the RULIT variables, some variables did not pass the assumption, and a non-parametric test was performed. A Wilcoxon signed-rank test was applied to establish differences between the control day and before and after the acute exercise stress in the variables’ total correct scores and total step errors. A parametric *T*-test was applied for the variables Trials, Performance and Memory recall. Additionally, to determine the effect of acute exercise on learning curves, repeated measures of ANOVA were conducted (2 acute exercise (pre and post) × 3 RULIT trial blocks (R2 to R4 (B1), R5 to R7 (B2), and R8 to R10 (B3))). Moreover, other repeated measures of ANOVA were also carried out (two successes followed (correct scores followed before and after first error) × 2 acute exercises (pre and post)). On all ANOVA analyses, pairwise comparison was carried out using Bonferroni correction. Finally, Spearman’s correlations were performed in order to establish the lineal relationships between CORT and the RULIT learning test variables. The effect size of the differences was calculated (*r* value). The level of significance was set at *p* < 0.05 for all analyses.

## Results

The maximal dynamic strength (1RM) corresponded to 151.3 ± 19.5 kg obtained through a test of 3.6 ± 1.9 repetitions. The loads displaced were : 77.8 ± 11.8 kg for Pmax −15%, 101.1 ± 14.0 kg for Pmax and 123.4 ± 16.8 kg for Pmax +15%, corresponding to 51.5 ± 5.3%, 66.9 ± 5.4% and 81.6 ± 5.2% of 1RM, respectively.

### Cortisol time-course

Further CORT-related analyses revealed that CORT basal C1 Familiarisation Day (Cbf) measures differed significantly between the CORT basal C1 Experimental Day (Cbe) (Cbc = 5.65, SE = 0.59; Cbe = 3.56, SE = 0.24), *t*(18) = 3.79, *p* < 0.001), which indicates relatively high levels of stress for the group on the Familiarisation Day. Additionally, Post-Cognitive CORT C2 Control Day (Pcf) measures differed significantly between the Post-Cognitive CORT C2 Experimental Day 1 (Pce; Pcc = 4.90, SE = 0.65; Pce = 3.29, SE = 0.33), *t*(18) = 3.21, *p* = 0.01).

**Figure 2:**
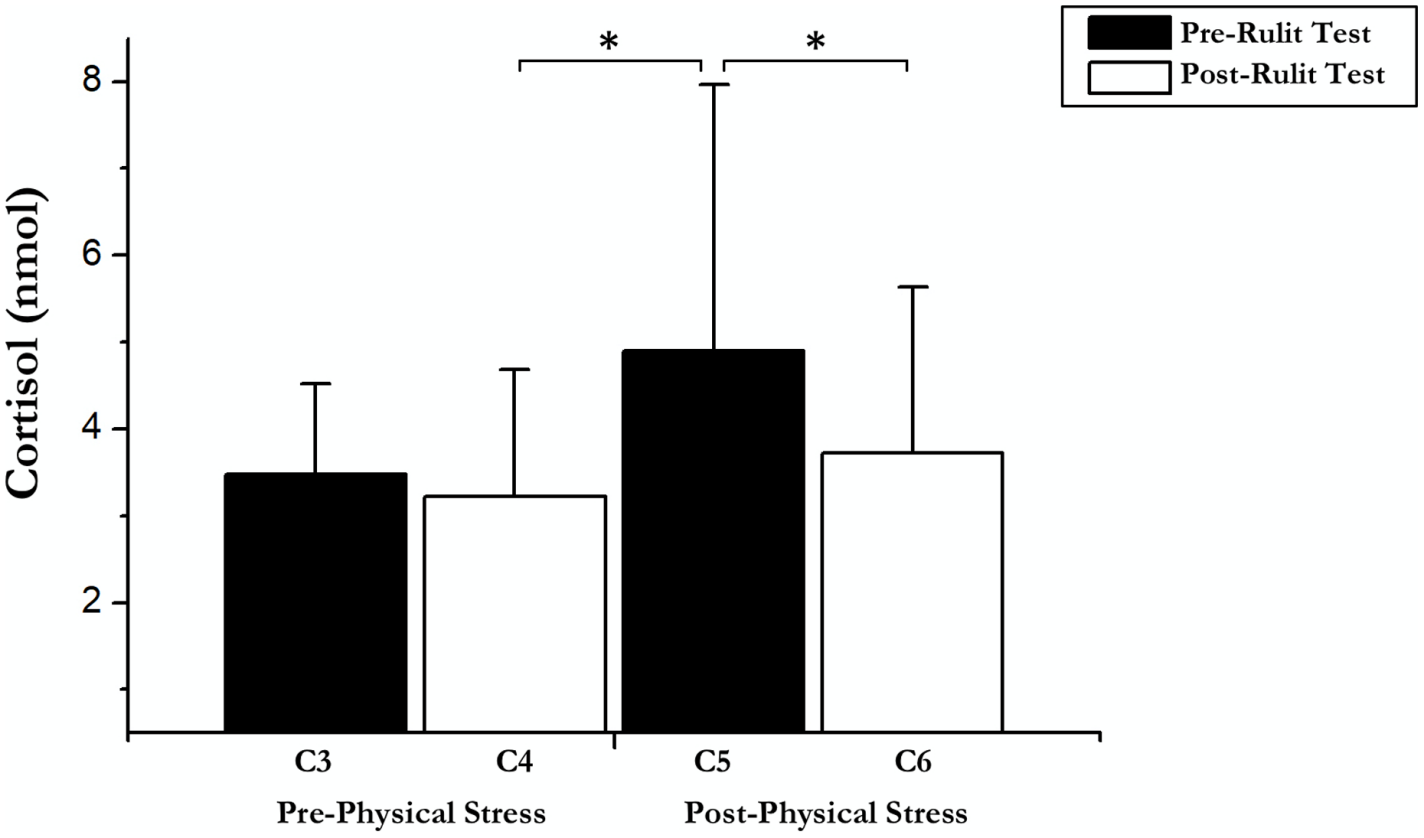
Cortisol data before and after exercise and RULIT test. An asterisk (*) indicates significant differences (*p* < 0.05).

On the other hand, an immediate influence of acute exercise on cortisol was found; a *T*-test showed significant differences between Post-Physical Stress C5 and C4 Post-Cognitive CORT (*t*(18) = –2.42; *p* = 0.026; *r* = 0.25) and C6 Post-Cognitive CORT in Experimental Day (*t*(18) = 4.04; *p* = 0.01; *r* = 0.48). Pairwise comparisons and *t*-test results are shown in Fig. 2.

Contrary to expectations, no correlations were found between the RULIT learning test variables evaluated and CORT reactivity under any condition.

### Ruff–Light trail-learning test

#### Learning variables

For the RULIT scores (Table 1), a Wilcoxon signed-rank test revealed (Familiarisation Day *vs*. Experimental Day) a significant effect for: total correct scores (*z* = –3.14; *p* = 0.002; *r* = –0.74), total steps errors (*z* = –3.31; *p* = 0.001; *r* = –0.78), performance (*t*(18) = –3.99; *p* < 0.001; *r* = 0.55) and trials (*t*(18) = 2.14; *p* = 0.046; *r* = 0.45). Accordingly, the Familiarisation Day (*vs*. Experimental Day) exhibited fewer correct steps, a higher error rate, and a significantly negative performance index for completing the path.

**Table 1:** Differences between pre and post-acute exercise in RULIT task.

	RULIT pre-acute exercise	RULIT post-acute exercise
Total correct scores	122.4 (3.25)	120.1 (5.93)*
Total step errors	4.2 (3.93)	6.5 (4.64)*
Performance	12.6 (0.93)	11.8 (0.98)*
Trials	4.6 (1.92)	4.8 (1.48)

**Notes:**

The data are expressed as mean (standard error).

An asterisk (*) indicate significant differences related to pre-acute exercise (*p* < 0.05).

On the other hand, for the visuo-spatial learning outcomes on the Experimental Day, there were significant differences between pre- and post-acute exercise in the RULIT learning variables, total correct scores (*z* = –2.26; *p* = 0.024; *r* = –0.52), total step errors (*z* = 2.46; *p* = 0.014; *r* = 0.56) and performance (*t*(18) = 6.1; *p* = 0.01; *r* = 0.8) (Table 1).

#### Learning curves

There were some main effects of the RULIT trial blocks (*F* (2, 36) = 84.3, *p* < 0.001, partial η^2^ = 0.82) on learning. Moreover, exercise-induced stress × RULIT trail block interaction (*F* (2, 36) = 3.67, *p* = 0.035, partial η^2^ = 0.17) was found. Pairwise comparisons of the Learning Curves are shown in Fig. 2.

#### Successes followed (working memory)

**Figure 3:**
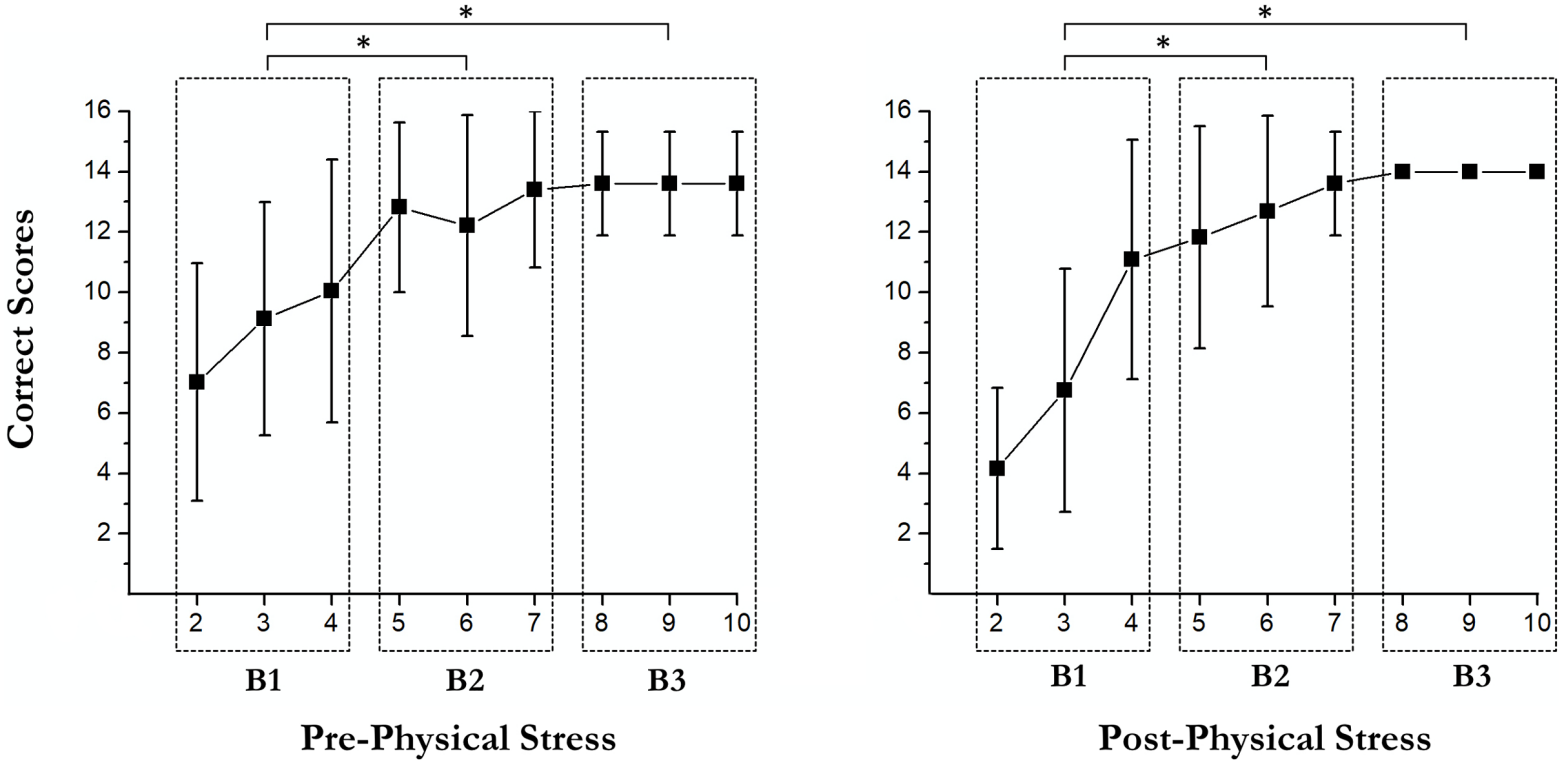
Pairwise comparisons of the learning curves. An asterisk (*) indicates significant differences between blocks (*p* < 0.05).

Our results showed a significant main effect of successes followed (*F* (1, 18) = 18.33, *p* < 0.001, partial η^2^ = 0.505), showing an increase in correct scores after the first error (*M* = 7.3; SD = 0.4) respect before (*M* = 5.4; SD = 0.6). Moreover, a significant main effect of exercise-induced stress was observed (*F* (1, 18) = 15.49, *p* = 0.001, partial η^2^ = 0.462). Specifically, in post-physical stress (*M* = 5.15; SD = 0.3) the scores were lower than under the pre-physical stress condition (*M* = 7.5; SD = 0.7); Fig. 3. No significant interaction between successes followed × exercise-induced stress was found (*F* (1, 18) = 1.31, *p* = 0.27, partial η^2^ = 0.068).

#### Delayed recall

**Figure 4:**
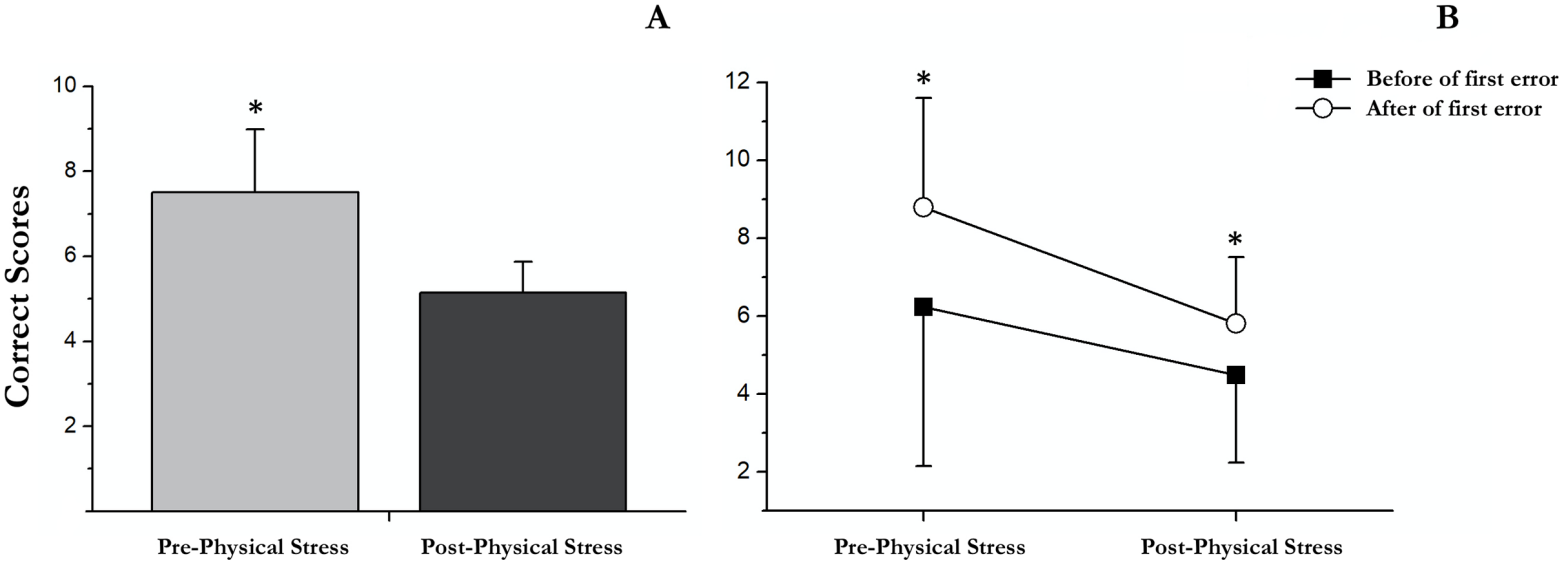
Correct scores followed before and after the first error in pre and post physical exercise. (A) An asterisk (*) indicates significant differences between pre and post physical exercise (*p* < 0.05). (B) An asterisk (*) indicates significant differences between correct scores followed before and after the first error (*p* < 0.05).

There were significant differences between pre- and post-acute exercise in RULIT *delayed recall* scores (*t*(18) = 4.83; *p* < 0.001; *r* = 0.7). Concretely, the successes were superior before (mean = 7.3, SD = 3.2) than after (mean = 4.6, SD = 1.5) acute exercise (Fig. 4).

## Discussion

The present study investigated CORT reactivity and visuo-spatial path learning under physical stress. It was assumed that performing a single bout of acute resistance exercise would provoke physical stress and trigger substantial CORT responses (Heijnen et al., 2016). Subsequently, assuming that high post-stress CORT responses affect the hippocampus and retrosplenial brain areas, an impairment in visuo-spatial learning and memory capacity would be expected.

The current findings confirmed that performing a single bout of acute resistance activates the hypothalamic–pituitary–adrenal (HPA) axis, leading to substantial differences in CORT concentrations between both conditions (pre- *vs*. post-acute stress exercise). In addition, post-acute stress exercise, participants showed worse performance in their visuo-spatial learning task (as expressed by fewer correct path steps and increased error rates and trail numbers) and memory (WM and delayed recall) capacity. Furthermore, with respect to the learning curves, slower learning to completion under stress conditions was found in the initial learning trials.

CORT concentrations differed significantly immediately after the acute exercise. This effect was expected and is in line with findings from previous research that showed strong endocrine and psychophysiological effects during and immediately after acute exercise, due to the stress that intense exercise generates in the body; the HPA axis is activated by increasing the level of cortisol (Heijnen et al., 2016). This increase can last several hours after intense or exhausting exercise (Willoughby, Taylor & Taylor, 2003). The results of this study show that subjects showed significantly higher levels of cortisol after an intense resistance exercise session. This increase in cortisol has been shown to be one of the causes of changes in cognition after intense physical exercise (Wang et al., 2019).

Single bouts of exercise increase, and regular exercise decreases, the oxidative challenge to the body, whereas excessive exercise and overtraining lead to damaging oxidative stress and thus are an indication of the other end point of the hormetic response (Radak et al., 2008). This means that, to obtain positive effects through a stressor, we must apply it in low doses because, if it is over-applied, it would cause harmful consequences to the body. Acute resistance exercise impaired cognitive performance, which supports the results from previous work that investigated post-stress visuo-spatial path-learning efficacy (Branaghan et al., 2010; Taverniers et al., 2011) and memory (Almela et al., 2012; Guenzel, Wolf & Schwabe, 2014). In this sense, Gathercole & Alloway (2008) have reported that the capacity of working memory is closely related to learning ability.

In addition, higher levels of cortisol were observed on the familiarisation day than on the experimental day (with a 48 h difference). Taking as a reference the four contextual factors of Lupien et al.’s (2009) experience of psychological stress—the effect of unpredictability, loss of control, novelty and threat to the ego—it appears that an unknown environment (Schnell et al., 2013) and the threat of cognitive-capacity assessment (Ennis et al., 2001) caused increased cortisol reactivity in response to stress. This is an important factor to take into account, since any investigation could be affected if it is carried out on a single day because the levels of excitability should not be controlled.

Neuropsychological findings and functional magnetic resonance imaging (fMRI) studies have confirmed the essential role of the hippocampus in human topographical mapping (*e.g*., the right hippocampus is strongly associated with knowledge of a spatial location and with navigating accurately between specific locations; Bohbot, Iaria & Petrides, 2004; Ekstrom et al., 2003; Maguire, 2001). This is why high post-stress CORT responses after exercise affect the hippocampus and retrosplenial brain areas, impairing visuo-spatial learning and memory-recall capacity.

Our results seem to suggest that these brain areas that were assessed with cognitive tasks were affected by high CORT concentrations. This assumption, however, is merely speculative, because no correlation has been found between cognitive-task performance and CORT concentrations. This lack of correlation could be attributed to the broad dispersion of salivary CORT responses to between the individual participants (Kudielka, Hellhammer & Wüst, 2009) and would be in line with other researchers (Deinzer et al., 1997; Taverniers et al., 2011) who found as many different CORT response patterns as subjects assessed, thus making this correlation difficult. Even so, several studies have linked decreases in cognitive performance when testing coincides with the peak of cortisol (Bermejo et al., 2021) and increases in cognitive performance to acute decreases in cortisol levels (Heaney, Carroll & Phillips, 2013; Tsai et al., 2014). There seems, then, to be a cause–effect relationship.

Another explanation of the results would come from the term *hormesis* (Calabrese et al., 2007), defined as an adaptive response of cells and organisms to a moderate (usually intermittent) stress. Examples include ischemic preconditioning, exercise, dietary-energy restriction and exposures to low doses of certain phytochemicals (Mattson, 2008). This dose-response phenomenon, characterised by stimulation at low doses and inhibition at high doses (bouts of acute exercise), results in an inverted-J- or U-shaped response curve to new doses.

The limits of the hormetic response are determined by the ability to adapt, or so-called “plasticity.”

According to arousal theory, the relationship between stress and activation follows an inverted “U” function (Joëls, 2006), which also occurs with other cognitive variables that depend to a greater or lesser extent on such activation. According to this function, while moderate stress levels cause cortisol reactivity and a moderate increase in cortisol secretion, which usually have a positive effect on cognitive performance (Guenzel, Wolf & Schwabe, 2014), very high levels of stress often lead to large increases in cortisol secretion associated with significant reductions in performance of different cognitive functions (Morgan et al., 2006, 2011; Taverniers et al., 2011; de Veld, Riksen-Walraven & de Weerth, 2014). In this regard, Tsai et al. (2014) found higher levels of cortisol after intense resistance exercise than after intense aerobic exercise.

It is, therefore, not surprising that there remains so much controversy in the literature relating acute exercise to cognitive performance, due mainly to the heterogeneity of the exercise protocols proposed in both the type of exercise and its intensity, as well as the diversity of tests that measure cognitive performance and all the variables related to it (Loprinzi, Loenneke & Storm, 2021).

In addition, the time elapsed between the cessation of exercise and the evaluation of cognitive functions is another crucial variable due to the transience of the psychophysiological effects of acute exercise (Crabbe & Dishman, 2004). Depending on the intensity, duration and rest intervals, resistance exercise can strongly stimulate the HPA axis, which can lead to an increase in adrenocorticotropic hormone (ACTH) and circulating cortisol (Kraemer et al., 1993; Kraemer et al., 1999, 1998).

Having noted all this, and focusing on the cognitive variables measured in this study, in terms of memory, different studies have shown that memory improves after intense aerobic exercise (Frith, Sng & Loprinzi, 2017; Jeon & Ha, 2017). As mentioned above, however, there is some controversy in the literature about this, as some authors have not found improvements in memory related to intense exercise or have found even some deterioration in memory (Guenzel, Wolf & Schwabe, 2014; Taverniers et al., 2011). Most existing publications regarding the effect of intense exercise on memory have considered the aerobic exercise (Chang et al., 2012). Far fewer studies have examined the effect of bouts of acute resistance exercise on memory (Wilke et al., 2019), and there remains controversy over the results. Many authors have found improvements in working memory after acute resistance exercise (Chang et al., 2014; Hsieh et al., 2016; Wu et al., 2019), while other researchers have studied the ideal exercise doses, so that there are positive results ruling out maximum or sub maximum exercises (Brush et al., 2016; Wilke et al., 2019).

There is considerable evidence that cortisol responses influence memory, and a number of models have proposed that the cortisol response is critically involved in producing the observed memory effects (Joëls, Fernandez & Roozendaal, 2011; Schwabe et al., 2012; Gagnon & Wagner, 2016). The main pathway claimed is an increase in glucocorticoids *via* the activation of the HPA axis (Allen et al., 2014; McEwen, 2007). In the same line, studies with animals have shown that glucocorticoids can exert causal influences on memory (de Quervain, Roozendaal & McGaugh, 1998; Roozendaal, 2002). Furthermore, cortisol administration independently influences memory encoding and retrieval (Het, Ramlow & Wolf, 2005).

In a meta-analysis by Shields et al. (2017), the effects of stress on cortisol did not predict effects on memory during any memory phase, indicating that stress may also act through pathways other than cortisol to influence memory.

Loprinzi et al. (2020) assessed memory function using a multiple trial, word-list episodic memory task (the Rey Auditory Verbal Learning Test, RAVLT), then performed a comprehensive, computerised assessment of episodic memory (the Treasure Hunt task, THT), which involved a spatio-temporal assessment of the identify, location and timing of components of episodic memory. The results demonstrated that acute high-intensity resistance exercise may impair episodic memory when a short exercise recovery period is employed, but with a longer recovery period, acute high-intensity resistance exercise may potentially enhance episodic memory (Loprinzi et al., 2020).

The results of this study reinforce the different publications that have concluded that acute resistance exercise at a certain intensity does not lead subjects to obtain better results on memory tests (Brush et al., 2016; Chang & Etnier, 2009); in our study, participants’ scores declined after exercise. The study’s limited capacity for information processing and memorisation is well known, so disproportionate storage or excessive demands can have serious consequences for ongoing cognitive activities (Alloway et al., 2009) or the ability to filter relevant and irrelevant information (Awh & Vogel, 2008). This capacity is limited by approximately 5–9 elements (7 ± 2, Miller’s magic number, 1956). This standard error of ±2 is reflected in the significant differences of Successes followed (WM) and the exercise’s effects on this variable. Thus, we can see that, after exercise, the manipulation of the information (WM) necessary for the achievement of the learning task is limited.

As for the relationship between intense exercise and learning ability, far fewer authors have studied this (Winter et al., 2007; Frith, Sng & Loprinzi, 2017). Some evidence, however, has been found that intense aerobic exercise can aid concentration and improve learning (Chang et al., 2014; Coles & Tomporowski, 2008). It must be highlighted that there exist very few studies that have analysed the influence of intense resistance exercise on learning (Chang et al., 2012; Wilke et al., 2019). Our results showed that, after an intense resistance exercise session, subjects showed a lower learning ability than before the session.

As described previously, this relationship may be due to the high intensity of the exercise protocol proposed in this study, as well as the waiting time between the performance of the physical exercise and the cognitive test carried out. It would be necessary, however, to analyse whether lower intensities of resistance exercise have the same effects on memory capacity and learning.

On the other hand, we should not forget that working memory enables the temporary storage and manipulation of the information needed to perform complex cognitive tasks, such as language understanding, learning and reasoning (Gathercole et al., 2006).Therefore, the deterioration of this factor (WM) should be expected to affect any associated task (learning in our case) irreparably.

Related to the cortisol time-curse, there was a significant reduction in cortisol after the cognitive tests in both conditions (pre- and post-exercise). From this fact it can be deduced that the cognitive task used in this study is not sufficient to maintain or increase cortisol (Veltman & Gaillard, 1998). Usually, the maximum peak of cortisol is 0–20 min after exercise and cortisol returns to pre-stressor levels by 41–60 min after the end of the stressor (Dickerson & Kemeny, 2004). The trend in our case would be to recover the initial cortisol levels.

This trend is of utmost importance, especially knowing that the majority of cortisol receptors are found in the hippocampus, an area fundamental to learning and memory (Elzinga & Bremner, 2002; Shin & Liberzon, 2010). A proper cortisol response is crucial for the physiological allostasis, whereas high loads of CORT are more likely to lead to the development of metabolic and/or brain disorders (Duman, 2002; Eiden, 2010). Numerous studies have documented the role of brain-derived neurotrophic factor (BDNF) in supporting learning and memory; Molteni, Ying & Gómez-Pinilla (2002) suggest that hippocampal levels of BDNF may be directly related to learning efficiency and memory stability. BDNF and cortisol play complementary roles in the nervous system, where cortisol is a regulator of positive/negative effects (de Assis & Gasanov, 2019). Secondly, resistance exercise supports the reduction in IL-6, which inhibits NMDA activity and increases in IGF-1 production (Loprinzi et al., 2020), both being determining factors in possible changes in cognition (Chang et al., 2012).

Finally, another important point to consider is the resistance exercise protocol used; working with multi-joint resistance exercises that vary the degree of balance required to complete the task increases cognitive demand (Loprinzi, Loenneke & Storm, 2021), which can lead to a limitation of the resources available after exercise.

## Conclusions

The study highlights the importance of load-, volume- and intensity-resistance exercise for better HPA activation control and, consequently, cortisol production, which could impair cognitive performance. This does not necessarily mean that resistance exercise will always have the same effect, as results may vary widely depending on the intensity and type of exercise, the moment of measurement and the cerebral areas implicated.

## Limitations

The experimental design and the technical capabilities of the researchers did not allow for use to address the effects of catecholamines or the interruption of cerebral oxygenation, physiological parameters that can also affect cognitive performance when performing intense acute exercise.

## Supplemental Information

10.7717/peerj.13000/supp-1 Supplemental Information 1 Raw data.

## Additional Information and Declarations

### Data Availability

The following information was supplied regarding data availability: The raw data are available in the Supplemental File.
